# Effects of Replacing Whole-Crop Maize Silage with *Pennisetum giganteum* Silage on the Rumen Microbial Community in Beef Cattle

**DOI:** 10.3390/ani16101535

**Published:** 2026-05-17

**Authors:** Shuai Fang, Junyu Zhang, Xubiao Han, Mirizhati Aireti, Yong Tuo, Bayin Bate, Meiling Yan, Kailibinuer Abudukaiyoumu, Tongjun Guo

**Affiliations:** 1 Xinjiang Uygur Autonomous Region Academy of Animal Sciences, Urumgi 830011, China; fangshuai1011@139.com (S.F.); 15709516935@139.com (X.H.); 13999422641@163.com (M.A.); t777yvx@126.com (Y.T.); 13899868383@163.com (B.B.); 15134708791@163.com (M.Y.); 17767554842@163.com (K.A.); 2 College of Animal Science, Xinjiang Agricultural University, Urumqi 830052, China; 3 Xinjiang Key Laboratory of Herbivorous Livestock Feed Biotechnology, Urumqi 830011, China

**Keywords:** *Pennisetum giganteum* silage, beef cattle, rumen microbiota, rumen fermentation

## Abstract

This study evaluated the effects of replacing whole-crop maize silage with varying proportions of *Pennisetum giganteum* silage on rumen fermentation, microbial composition, and metabolic function in beef cattle. A single-factor completely randomized design was employed using 50 healthy crossbred Simmental cattle aged 11–12 months (average body weight: 251.08 ± 51.54 kg). Animals were randomly assigned to five groups, with 10 replicates per group and one animal per replicate. Diets contained 0% (Group A), 25% (Group B), 50% (Group C), 75% (Group D), or 100% (Group E) *Pennisetum giganteum* silage replacing whole-crop maize silage over a 67-day feeding period, including a 7-day adaptation phase. Rumen fluid samples were collected via rumen catheter at the end of the trial to assess bacterial diversity and functional characteristics. Increasing the proportion of *Pennisetum giganteum* silage resulted in quadratic changes in volatile fatty acids (VFAs) and propionate (PA) concentrations (*p* < 0.05), while ammonia nitrogen (NH_3_-N) increased linearly (*p* < 0.05). No significant differences were observed in α- or β-diversity among groups (*p* > 0.05). Group C exhibited significantly higher relative abundances of Verrucomicrobiota and *Prevotellaceae_UCG_003* compared with the other groups (*p* < 0.05). At the phylum level, Proteobacteria increased linearly, whereas Spirochaetota decreased linearly; at the genus level, *Treponema* decreased linearly (*p* < 0.05). LEfSe analysis indicated enrichment of *g__Prevotellaceae_UCG_003* and o__WCHB1_41 in Group C, while the relative abundances of f__Enterobacteriaceae and *g__Citrobacter* were elevated in Group E. Under the conditions of this study, replacing 50% of whole-crop maize silage with *Pennisetum giganteum* silage enhanced rumen fermentation efficiency and modulated key microbial populations in beef cattle.

## Simple Summary

In a 67-day feeding trial with beef cattle, whole-crop maize silage was replaced with *Pennisetum giganteum* silage at substitution ratios of 0%, 25%, 50%, 75%, and 100%. Changes in the rumen microbial composition of beef cattle were further analyzed to evaluate the effects of different diets on the rumen environment. The results showed that replacing whole-crop maize silage with 50% *Pennisetum giganteum* silage improved rumen fermentation parameters and modulated key Microbial groups. These findings indicate that the appropriate inclusion of *Pennisetum giganteum* silage may serve as a promising alternative feed resource in beef cattle production.

## 1. Introduction

The rumen microbiota comprises bacteria, archaea, protozoa, fungi, and viruses (primarily bacteriophages) and performs essential functions, including proteolysis, fiber degradation, and lipid hydrolysis. Under anaerobic conditions, these microorganisms ferment dietary carbohydrates and proteins to produce VFAs, microbial protein, peptides, and amino acids [1], thereby supplying more than 70% of the host’s energy and approximately 80% of its protein requirements [2,3]. Variations in the rumen microbiota are closely associated with multiple host phenotypes, including feed efficiency and health status [4,5,6].

In beef cattle production systems, high-quality forage is the material basis for maintaining normal rumen fermentation and improving productive performance. Whole-crop maize silage has been widely used in finishing cattle production because of its high palatability, relatively high energy value, and stable fermentation quality [7,8]. However, in some regions, the consistent supply of whole-crop maize silage is constrained by limitations in arable land resources, climatic conditions, irrigation availability, and cultivation costs, which in turn restricts the sustainable development of the beef cattle industry [9,10].Therefore, the development of alternative forage resources characterized by high yield, superior quality, and strong environmental adaptability has become an important focus of ruminant nutrition research.

*Pennisetum giganteum*, native to Africa, is a perennial high-yield gramineous forage characterized by rapid growth, high biomass production, strong regrowth capacity, excellent stress tolerance, and broad environmental adaptability [11,12,13]. The plant generally reaches a height of 3–5 m, with a maximum recorded height of 7.0 8 m. After 4 weeks of growth, the annual fresh biomass yield can reach 200–400 t/ha, with a crude protein content of 10.8% [14]. Previous studies have shown that replacing maize silage with a mixed silage containing 50% *Pennisetum giganteum* and rice straw in finishing lamb diets improved fiber digestibility and enhanced antioxidant capacity [15]. In addition, *Pennisetum giganteum* silage has been reported to improve gastrointestinal microbial homeostasis in goats, alleviate heat stress, and enhance antioxidant status and immune function [16]. Furthermore, *Pennisetum giganteum* silage increased the apparent digestibility of Neutral Detergent Fiber (NDF) and Acid Detergent Fiber (ADF) in beef cattle and can serve as a major roughage source in beef production systems [17]. Although previous studies have demonstrated the application potential of *Pennisetum giganteum* in ruminants, the effects of replacing whole-crop maize silage with *Pennisetum giganteum* silage on rumen fermentation patterns and rumen microbiota structure have not been fully elucidated. It remains unclear whether different substitution ratios of *Pennisetum giganteum* silage for whole-crop maize silage alter the composition of dominant rumen microbial taxa, microbial diversity, and the formation patterns of fermentation end products.

Therefore, the present study applied a graded substitution strategy in which whole-crop maize silage was progressively replaced with *Pennisetum giganteum* silage. The objective was to systematically evaluate its effects on rumen fermentation parameters, rumen microbiota structure, and microbial diversity in beef cattle, and to elucidate the potential mechanisms by which *Pennisetum giganteum* silage modulates rumen function. The findings of this study provide a scientific basis and practical reference for the rational utilization of *Pennisetum giganteum* silage in beef cattle production and for optimizing ruminant diet formulation.

## 2. Materials and Methods

All animal care and handling procedures in this study were conducted in strict accordance with the Guidelines for the Care and Use of Laboratory Animals in China and were approved and supervised by the Animal Care and Use Committee of the Xinjiang Academy of Animal Sciences (Approval No. XKYSLS-2024-09; Urumqi, China).

### 2.1. Experimental Materials

The *Pennisetum giganteum* silage and whole-crop maize silage used in this experiment were obtained from a livestock farm located in Shangsan Gong Village, Hutubi County. Their nutrient compositions are presented in Table 1.

### 2.2. Animals and Experimental Design

The experiment was conducted from November 2024 to January 2025 at a livestock farm located in Shangsan Gong Village, Hutubi County, Changji Prefecture, Xinjiang, China. A total of 50 healthy fattening Simmental crossbred beef cattle (initial body weight: 251.08 ± 51.54 kg) were selected and randomly assigned to five groups, with 10 animals per group. Diets were formulated according to the Feeding Standard of Beef Cattle (2004) [18], based on isoenergetic and isonitrogenous principles, to achieve an Average Daily Gain (ADG) of 1.0 kg. The dietary composition and nutrient levels are presented in Table 2. The replacement levels of *Pennisetum giganteum* silage for whole-crop maize silage were 0% (Group A), 25% (Group B), 50% (Group C), 75% (Group D), and 100% (Group E). The experimental period lasted 67 days, including a 7-day adaptation period and a 60-day formal trial period.

All animals were housed in a tie-stall system and fed twice daily at 10:00 and 19:00, with ad libitum access to feed and water. Disinfection and immunization procedures were carried out according to standard farm management practices. To minimize pen effects, all experimental pens were located in adjacent areas within the same row, ensuring consistent environmental conditions, including lighting, ventilation, temperature, and humidity. A standardized management protocol was implemented, with the same personnel responsible for feeding, manure removal, and water provision to ensure uniformity across groups. There were no significant differences in initial body weight among groups (*p* > 0.05), indicating good comparability at the start of the experiment.

### 2.3. Sample Collection and Measurement of Parameters

#### 2.3.1. Collection of Rumen Fluid

On the final day of the experiment, six cattle were randomly selected from each group for rumen fluid collection. All samples were obtained using an oral stomach tube. Prior to sampling, animals were gently restrained in a standing position to minimize movement and ensure operator safety. All procedures were performed by trained personnel to reduce handling time and stress. The oral stomach tube was lubricated and carefully inserted through the oral cavity into the rumen, with care taken to ensure correct placement and avoid tracheal insertion. The sampling procedure was completed as quickly as possible, and animals were immediately returned to normal feeding conditions after collection. The initial portion of rumen fluid (approximately 50 mL) was discarded to avoid saliva contamination, and the subsequent rumen fluid was retained for analysis. The rumen pH was measured immediately using a portable pH meter (PHS-3C, Shanghai, China). Subsequently, the samples were aliquoted into 15 mL centrifuge tubes and 5 mL cryovials. The samples in the 15 mL centrifuge tubes were stored at −20 °C for the determination of NH_3_-N and VFAs concentrations. The samples in the 5 mL cryovials were immediately frozen in liquid nitrogen and stored for subsequent analysis of the rumen microbial community.

#### 2.3.2. Determination of Rumen Fermentation Parameters

After thawing, the samples were centrifuged at 4 °C and 1000× *g* for 10 min to obtain the rumen supernatant for volatile fatty acid analysis. The concentrations of VFAs in rumen fluid were determined using a gas chromatograph (GC-2010, Shimadzu, Kyoto, Japan) according to the method described by Mohammed et al. [19]. The concentration of NH_3_-N was measured using the alkaline hypochlorite–phenol colorimetric method [20].

#### 2.3.3. Analysis of the Rumen Microbial Community

Total genomic DNA was extracted from 30 samples using the TGuide S96 Magnetic Soil/Stool DNA Kit (Tiangen Biotech Co., Ltd., Beijing, China) according to manufacturer’s instructions. The quality and quantity of the extracted DNA were examined using electrophoresis on a 1.8% agarose gel, and DNA concentration and purity were determined with NanoDrop 2000 UV-Vis spectrophotometer (Thermo Scientific, Wilmington, DE, USA). The hypervariable region V3–V4 of the bacterial 16S rRNA gene were amplified with primer pairs 338F: 5′-ACTCCTACGGGAGGCAGCA-3′ and 806R: 5′-GGACTACHVGGGTWTCTAAT-3′. Both the forward and reverse 16S primers were tailed with sample-specific Illumina index sequences to allow for deep sequencing. The PCR was performed in a total reaction volume of 10 μL: DNA template 5–50 ng, forward primer (10 μM) 0.3 μL, reverse primer (10 μM) 0.3 μL, KOD FX Neo Buffer 5 μL, dNTP (2 mM each) 2 μL, KOD FX Neo 0.2 μL, and finally ddH2O up to 20 μL. After with initial denaturation at 95 °C for 5 min, followed by 20 cycles of denaturation at 95 °C for 30 s, annealing at 50 °C for 30 s, and extension at 72 °C for 40 s, and a final step at 72 °C for 7 min. The amplified products were purified with Omega DNA purification kit (Omega Inc., Norcross, GA, USA) and quantified using Qsep-400 (BiOptic, Inc., New Taipei City, Taiwan, ROC). The amplicon library was paired-end sequenced (2 × 250) on an Illumina novaseq6000 (Beijing Biomarker Technologies Co., Ltd., Beijing, China).

The qualified sequences with more than 97% similarity thresholds were allocated to one operational taxonomic unit (OTU) using USEARCH (version 10.0). Taxonomy annotation of the OTUs/ASVs was performed based on the Naive Bayes classifier in QIIME2 [21] using the SILVA database [22] (release 138.1) with a confidence threshold of 70%. Alpha was performed to identify the complexity of species diversity of each sample utilizing QIIME2 (2020.6) software. Beta diversity calculations were analyzed by principal coordinate analysis (PCoA) to assess the diversity in samples for species complexity. One-way analysis of variance was used to compare bacterial abundance and diversity. Linear discriminant analysis (LDA) coupled with effect size (LEfSe) [23] was applied to evaluate the differentially abundant taxa. The online platform BMKCloud (https://www.biocloud.net, accessed on 25 March 2025) was used to analyze the sequencing data.

### 2.4. Statistical Analysis

All experimental data were compiled and organized using Microsoft Excel 2021. Statistical analyses were performed using SPSS version 26.0 (IBM Corp., Armonk, NY, USA). One-way analysis of variance (ANOVA) was conducted to evaluate differences among groups, followed by Duncan’s multiple range test for post hoc comparisons. The results are presented as means, and the standard error of the mean (SEM) was used to indicate variability among groups. Statistical significance was declared at *p* < 0.05.

## 3. Results

### 3.1. Effects of Pennisetum giganteum Silage on Rumen Fermentation Parameters in Beef Cattle

According to Table 3, the inclusion of *Pennisetum giganteum* silage in the diet induced a quadratic response in VFAs and (PA concentrations in the rumen (*p* < 0.05). NH_3_–N content increased linearly with the proportion of *Pennisetum giganteum* silage in the diet (*p* < 0.05). Specifically, NH_3_–N concentrations in groups C, D, and E were elevated by 88.94% and 80.17%, 82.30% and 73.84%, and 86.06% and 77.43%, respectively, compared with the control and group B (*p* < 0.05). No significant differences were observed for the other measured rumen fermentation parameters (*p* > 0.05).

### 3.2. Rumen Microbial Community and Functional Analysis

#### 3.2.1. Venn Diagram Showing Overlap of Rumen Bacterial Taxa Across Treatments

**Figure 1:**
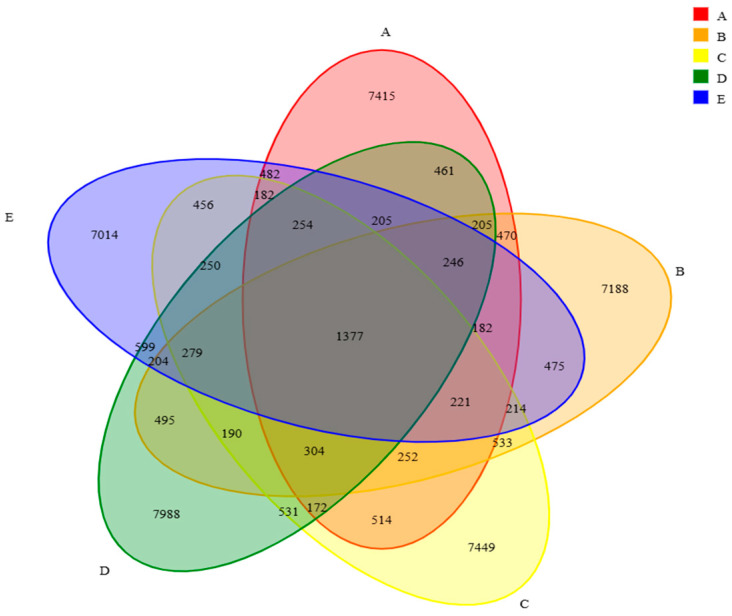
Venn diagram of rumen bacteria. Notes: Diets A, B, C, D, and E represented treatment groups in which whole-plant corn silage was replaced with *Pennisetum giganteum* silage at substitution levels of 0%, 25%, 50%, 75%, and 100%, respectively.

Figure 1 presents the Venn diagram of rumen bacterial communities in beef cattle fed diets in which *Pennisetum giganteum* silage replaced whole-plant corn silage at different proportions. A total of 2,400,113 paired-end reads were obtained from 30 samples. After quality filtering and assembly, 2,212,324 clean reads were generated. Each sample yielded at least 72,955 clean reads, with an average of 73,744 clean reads per sample. The numbers of operational taxonomic units (OTUs) in groups A to E were 7415, 7188, 7449, 7988, and 7014, respectively.

#### 3.2.2. Effects of *Pennisetum giganteum* Silage on the Alpha Diversity of the Rumen Microbiota in Beef Cattle

**Figure 2:**
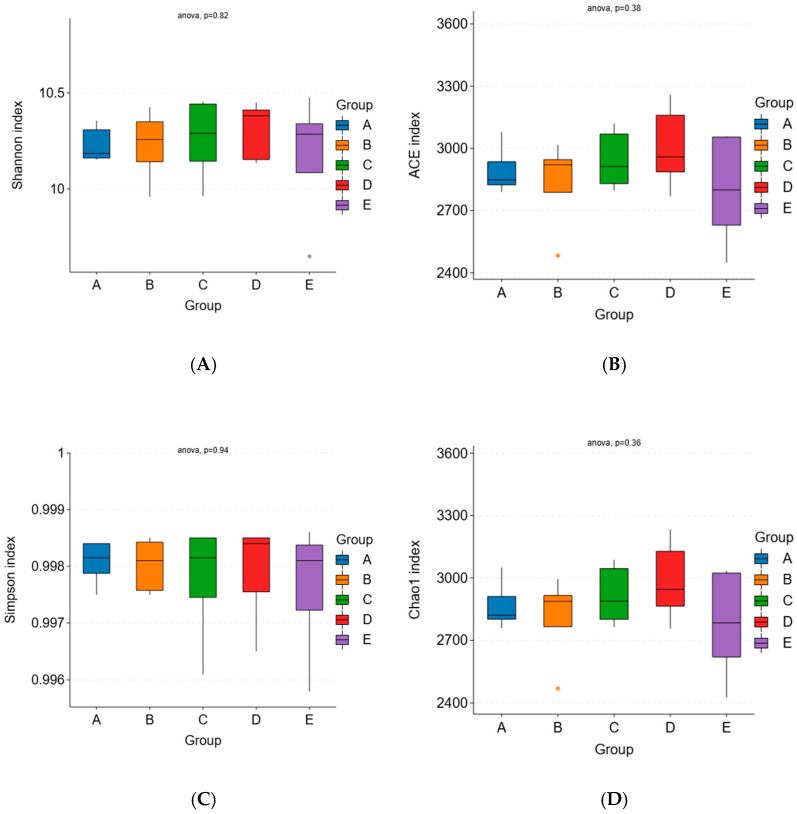
Effects of *Pennisetum giganteum* Silage on the Alpha Diversity of the Rumen Microbiota in Beef Cattle. Notes: Diets A, B, C, D, and E represented treatment groups in which whole-plant corn silage was replaced with *Pennisetum giganteum* silage at substitution levels of 0%, 25%, 50%, 75%, and 100%, respectively. (**A**) Boxplot of the Shannon index across different treatment groups; (**B**) Boxplot of the ACE index across different treatment groups; (**C**) Boxplot of the Simpson index across different treatment groups; (**D**) Boxplot of the Chao1 index across different treatment groups.

As shown in Table 4, the coverage indices for all groups exceeded 99%, indicating that the sequencing depth sufficiently captured the majority of species present in the samples. As illustrated in Figure 2, the Ace, Chao1, and Shannon indices in groups C and D were higher than those in group A. However, no significant differences were observed among groups for the Simpson, Shannon, Chao1, or Ace indices (*p* > 0.05).

#### 3.2.3. Effects of *Pennisetum giganteum* Silage on the Beta Diversity of the Rumen Microbiota in Beef Cattle

**Figure 3:**
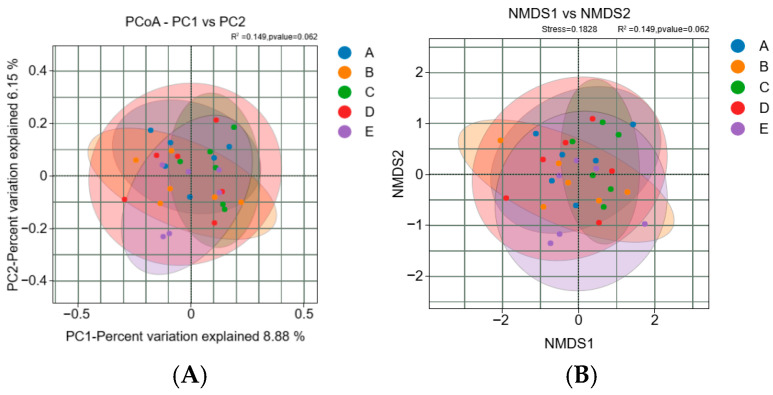
Beta diversity analysis of beef cattle rumen coordinates and metric multidimensional calibration analysis. (**A**) Principal Coordinate Analysis (PCoA) of microbial communities. (**B**) Non-metric multidimensional scaling (NMDS) of microbial communities. Notes: Diets A, B, C, D, and E represented treatment groups in which whole-plant corn silage was replaced with *Pennisetum giganteum* silage at substitution levels of 0%, 25%, 50%, 75%, and 100%, respectively.

Principal coordinates analysis (PCoA) and non-metric multidimensional scaling (NMDS) were performed based on the Bray–Curtis distance matrix (Figure 3A). The PCoA results showed a certain degree of overlap among samples from different treatment groups in the two-dimensional ordination space. The first two principal coordinates (PC1 and PC2) explained 8.88% and 6.15% of the total community variation, respectively. No clear separation trend was observed among treatment groups, indicating relatively small intergroup differences. According to the Adonis analysis, the differences among groups were not statistically significant (R^2^ = 0.149, *p* = 0.062).

The NMDS results were consistent with those of the PCoA (Figure 3B). Sample points from different groups were dispersed but largely overlapped, with no distinct clustering patterns observed. The stress value of the NMDS analysis was 0.1828, indicating an acceptable level of ordination reliability. Further statistical analysis also confirmed that there were no significant differences in the rumen microbiota structure among the treatment groups (R^2^ = 0.149, *p* = 0.062).

#### 3.2.4. Effects of *Pennisetum giganteum* Silage on the Composition of the Rumen Microbiota at the Phylum and Genus Levels

**Figure 4:**
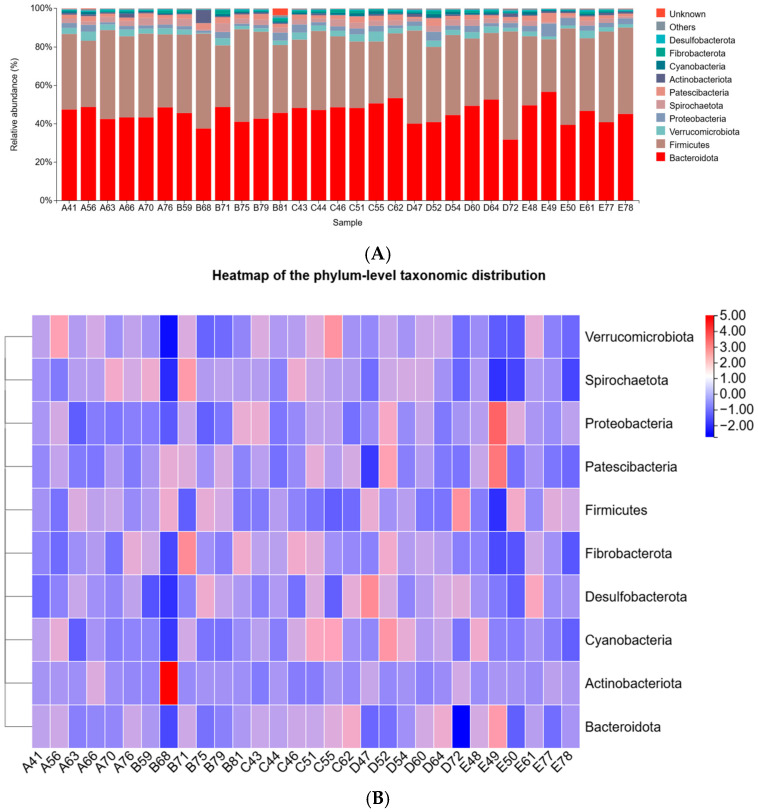
Rumen microbiota composition at the phylum level. (**A**) Bar plot showing the taxonomic relative abundance. (**B**) Clustered heatmap of the abundance profiles. Notes: Diets A, B, C, D, and E represented treatment groups in which whole-plant corn silage was replaced with *Pennisetum giganteum* silage at substitution levels of 0%, 25%, 50%, 75%, and 100%, respectively. The numbers following each letter designation (e.g., A41 and B59) indicate the individual identification numbers of the experimental beef cattle.

As shown in Figure 4, a total of 11 bacterial phyla were identified at the phylum level. According to Table 5, the dominant phyla across all groups were Bacteroidota and Firmicutes, accounting for 43.90–49.40% and 35.70–42.50%, respectively. The relative abundance of Bacteroidota in group C was higher than that in the other groups, whereas the relative abundance of Firmicutes was lower. Compared with group A, the relative abundance of Verrucomicrobiota was significantly decreased in groups B and E (*p* < 0.05), while no significant differences were observed in groups C and D (*p* > 0.05). The relative abundance of Proteobacteria increased linearly with increasing substitution of *Pennisetum giganteum* silage (*p* < 0.05), whereas Spirochaetota showed a linear decrease (*p* < 0.05).

**Figure 5:**
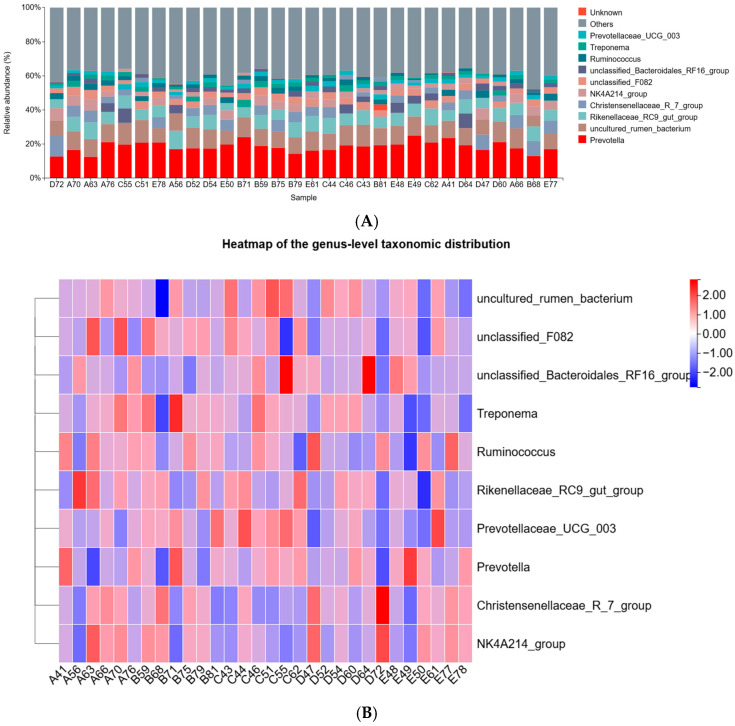
Rumen microbiota composition at the genus level. (**A**) Bar plot showing the taxonomic relative abundance. (**B**) Clustered heatmap of the abundance profiles. Notes: Diets A, B, C, D, and E represented treatment groups in which whole-plant corn silage was replaced with *Pennisetum giganteum* silage at substitution levels of 0%, 25%, 50%, 75%, and 100%, respectively. The numbers following each letter designation (e.g., A41 and B59) indicate the individual identification numbers of the experimental beef cattle.

As shown in Figure 5, the dominant genera in all groups were *Prevotella*, *uncultured_rumen_bacterium*, and *Rikenellaceae_RC9_gut_group*. As indicated in Table 6, the relative abundance of *Prevotellaceae_UCG-003* in group C was significantly higher than that in groups A, D, and E (*p* < 0.05), but did not differ significantly from group B (*p* > 0.05). The relative abundance of *Treponema* decreased linearly with increasing substitution of *Pennisetum giganteum* silage (*p* < 0.05), whereas no significant differences were observed among groups for the other genera (*p* > 0.05).

#### 3.2.5. LEfSe Species Difference Analysis

**Figure 6:**
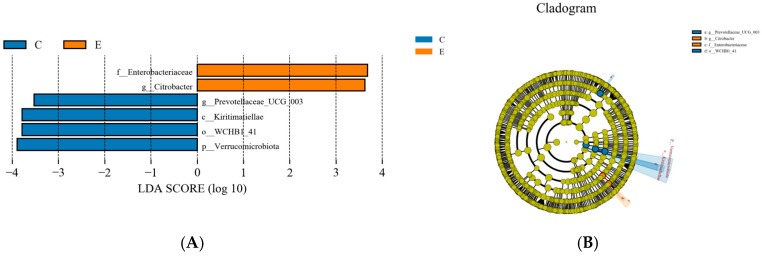
LDA score distribution among groups. LDA > 3.5, *p* < 0.05. (**A**) Biomarkers with LDA scores exceeding the threshold (LDA > 3.5), indicating statistically significant differences among groups. (**B**) Cladogram representing the phylogenetic distribution of taxa, in which concentric circles radiating outward denote taxonomic levels from kingdom (innermost ring) to genus (or species) (outermost ring). Notes: Diets C, and E represented treatment groups in which whole-plant corn silage was replaced with *Pennisetum giganteum* silage at substitution levels of 50% and 100%, respectively.

As shown in Figure 6A,B, Linear Discriminant Analysis Effect Size (LEfSe) was applied to identify differential features of the rumen bacterial communities among treatment groups, using a linear discriminant analysis (LDA) threshold > 3.5. The results indicated that *g__Prevotellaceae_UCG_003* and o__WCHB1_41 were enriched in the C treatment group, whereas the relative abundances of f__Enterobacteriaceae and *g__Citrobacter* were increased in the D treatment group.

#### 3.2.6. Associations Between Rumen Fermentation Parameters and Rumen Bacterial Communities

**Figure 7:**
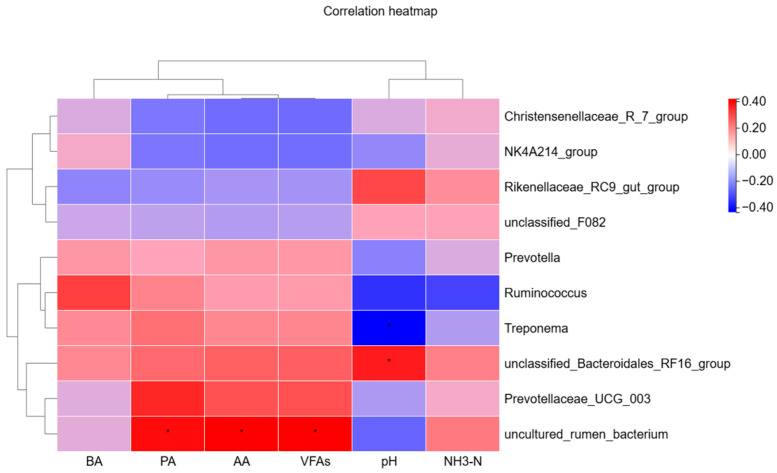
Relationships between the relative abundance of rumen bacteria and rumen fermentation parameters. * indicates *p* < 0.05. AA, Acetate; PA, propionate; BA,Butyrate; VFAs, volatile fatty acids; NH_3_-N, ammonia nitrogen.

Spearman’s rank correlation coefficients were calculated, and a heatmap was constructed to illustrate the relationships between rumen fermentation parameters and rumen bacterial taxa (Figure 7). The results showed that rumen pH was positively correlated with *unclassified_Bacteroidales_RF16_group* (*p* < 0.05) and negatively correlated with *Treponema* (*p* < 0.05). In addition, PA, Acetate (AA), and VFAs were positively correlated with *uncultured_rumen_bacterium* (*p* < 0.05).

#### 3.2.7. Rumen Bacterial Functional Prediction

**Figure 8:**
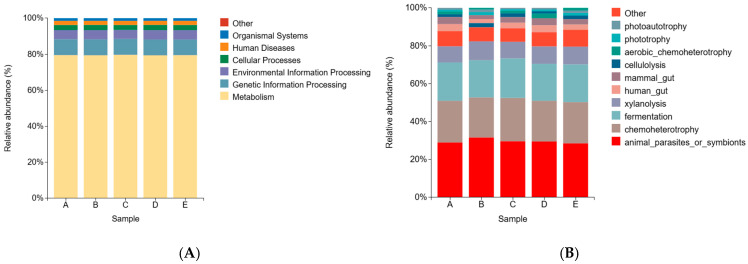
Functional Analysis of Rumen Microbiota (**A**) Functional prediction based on PICRUSt2 (Phylogenetic Investigation of Communities by Reconstruction of Unobserved States, version 2). (**B**) Functional inference based on FAPROTAX (Functional Annotation of Prokaryotic Taxa). Notes: Diets A, B, C, D, and E represented treatment groups in which whole-plant corn silage was replaced with *Pennisetum giganteum* silage at substitution levels of 0%, 25%, 50%, 75%, and 100%, respectively.

As shown in Figure 8, functional predictions based on PICRUSt2 (Phylogenetic Investigation of Communities by Reconstruction of Unobserved States, version 2) and FAPROTAX (Functional Annotation of Prokaryotic Taxa) revealed that different inclusion levels of *Pennisetum giganteum* silage resulted in highly consistent rumen microbial functional profiles across all treatments. The PICRUSt2 analysis indicated that the predicted functions associated with metabolism, genetic information processing, and environmental information processing accounted for approximately 79%, 8%, and 5% of the total functions, respectively, across all groups.

In parallel, FAPROTAX-based functional inference demonstrated that the dominant ecological functions of the rumen microbiota in all groups were chemoheterotrophy, fermentation, and fiber degradation, including xylanolysis and cellulolysis. The relative abundances of these key functional categories remained stable across the different dietary treatments, suggesting that varying the proportion of *Pennisetum giganteum* silage did not markedly alter the overall functional potential of the rumen microbial community.

## 4. Discussion

### 4.1. Effects of Pennisetum giganteum Silage on Rumen Fermentation Parameters in Beef Cattle

Rumen pH, NH_3_-N, and VFAs are key indicators for evaluating the physiological metabolic status of the rumen microbiota and the extent of dietary fermentation in the rumen [24]. Rumen pH was maintained within the range of 5.5–7.5, which comprehensively reflects microbial metabolic activity as well as the dynamic balance among the production, absorption, outflow, and neutralization of organic acids [25]. No significant differences in rumen pH were observed among treatments, and all values remained within the normal physiological range, indicating that *Pennisetum giganteum* silage did not exert significant adverse effects on rumen fermentation in beef cattle. Previous studies have shown that an NH_3_-N concentration of 5–30 mg/dL is optimal for the growth and proliferation of rumen microorganisms [26]. Although all experimental diets were formulated to be isoenergetic and isonitrogenous, ruminal NH_3_-N concentration still showed a linear increase as the substitution level of *Pennisetum giganteum* silage increased, suggesting that different forage sources can influence nitrogen metabolism under relatively stable rumen fermentation conditions. This result may be associated with differences in protein composition and ruminal degradation rates between *Pennisetum giganteum* silage and whole-crop maize silage. As the inclusion level of *Pennisetum giganteum* silage increased, the release of rumen-degradable nitrogenous components in the diet may have increased, whereas microbial assimilation of ammonia did not increase proportionally, resulting in the accumulation of NH_3_-N in the rumen. This finding was consistent with the results reported by Fang et al. [15].

VFAs are the major end products of carbohydrate fermentation by rumen microorganisms and provide approximately 70–80% of the metabolizable energy required by ruminants. AA is mainly derived from fiber degradation and serves as an important precursor for lipid synthesis [27,28]. PA is the principal precursor for hepatic gluconeogenesis and can be converted into glucose, thereby supplying energy to the animal and promoting growth and metabolism [29]. In the present study, total VFAs and PA exhibited a quadratic increase, indicating that replacement with *Pennisetum giganteum* silage may promote rumen fermentation and improve energy supply efficiency. These results suggest that *Pennisetum giganteum* silage can simultaneously influence ruminal carbon and nitrogen metabolism, although the utilization efficiencies of these two nutrient sources may not be fully synchronized.

### 4.2. Rumen Microbial Community and Functional Analysis

Rumen microbiota play a critical role in converting indigestible feed components into utilizable nutrients, and their diversity and abundance are essential for maintaining normal physiological functions [30]. Alpha (α) diversity reflects the richness and diversity of microbial species within a sample, whereas beta (β) diversity primarily represents differences in microbial community composition among samples. In the present study, α-diversity analysis showed that dietary inclusion of *Pennisetum giganteum* silage at different levels had no significant effects on the Chao1, Ace, Shannon, or Simpson indices in the rumen of beef cattle (*p* > 0.05). Furthermore, principal coordinates analysis (PCoA) and non-metric multidimensional scaling (NMDS) indicated no statistically significant differences in bacterial community structure among the treatment groups (*p* > 0.05). Under the conditions of this study, neither α- nor β-diversity of the rumen microbiota differed significantly among treatments. These findings suggest that the inclusion of *Pennisetum giganteum* silage had limited effects on overall microbial richness, evenness, and community structure, and that the rumen microbial ecosystem remained relatively stable.

Studies have demonstrated that the dominant bacterial phyla in the rumen of ruminants are Firmicutes, Bacteroidota, and Verrucomicrobiota. These microbial groups play essential roles in the degradation of complex carbohydrates and in promoting nutrient breakdown and absorption by the host [31,32]. Among them, Bacteroidota and Verrucomicrobiota are primarily involved in the fermentation and degradation of cellulose, soluble sugars, and other carbohydrates, producing small-molecule metabolites such as VFAs that are utilized by the host [33]. In contrast, Firmicutes are mainly associated with enhanced lipid metabolism and improved energy absorption efficiency in the host [34]. Fujisaka et al. [35] reported that Firmicutes and Bacteroidota together account for approximately 70–90% of the total bacterial population. In the present study, the relative abundances of Firmicutes and Bacteroidota accounted for approximately 80% of the total bacterial community, which is consistent with previous findings. When 50% of whole-crop maize silage was replaced with *Pennisetum giganteum* silage, the relative abundances of Bacteroidota and Verrucomicrobiota increased. Combined with the increase in total VFAs and the elevated AA-to-PA ratio, these findings suggest that substitution with *Pennisetum giganteum* silage may enhance the utilization of fibrous substrates by rumen microorganisms and shift rumen fermentation characteristics toward a pattern more favorable for fiber utilization. At the genus level, *Prevotellaceae_UCG_003* plays a key role in the degradation and metabolism of complex substrates such as cellulose and protein. In addition, it may promote the growth and development of beef cattle through synergistic interactions with other rumen microorganisms [7,36]. Wei et al. [37] demonstrated that supplementation with Astragalus root extract in yaks increased the relative abundance of WCHB1-41, thereby improving final body weight and ADG. With increasing substitution levels of *Pennisetum giganteum* silage, Spirochaetota and its representative genus *Treponema* decreased linearly, whereas Proteobacteria increased linearly, indicating that changes in diet composition exerted sustained selective pressure on the rumen microbiota and promoted adaptive restructuring of the microbial community [38,39,40]. Meanwhile, the abundance of *Prevotellaceae_UCG_003* increased in group C, which may be associated with the higher fiber content and the increased availability of complex plant polysaccharide substrates. The increased abundance of Verrucomicrobiota further reflected the adaptive adjustment of the rumen microbial community to the new dietary structure. This finding was consistent with the results of the Linear Discriminant Analysis Effect Size (LEfSe) analysis, which identified the dominant taxa enriched in group C. Overall, these microbial shifts were generally consistent with our previous observations that the 50% substitution level was superior to the other treatment groups in terms of growth performance and nutrient digestibility in beef cattle [41]. Silage quality could also contribute to the observed microbial variations, and these parameters may be considered in future studies for a more comprehensive assessment.

Based on the correlation heatmap analysis between rumen fermentation parameters and bacterial taxa, uncultured rumen bacterium showed significant positive correlations with AA, PA, and VFAs, suggesting that this taxon may be involved in carbohydrate utilization or VFAs production [42,43], and may partially explain the increased PA and VFAs concentrations observed in group C. Meanwhile, rumen pH was positively correlated with the unclassified Bacteroidales RF16 group and negatively correlated with *Treponema*. However, no significant differences in rumen pH or the RF16 group were detected among treatments, whereas *Treponema* showed a linear decline with increasing substitution levels. These results indicate that the correlation between pH and *Treponema* more likely reflects covariation among samples rather than a direct regulatory effect of pH, and that changes in *Treponema* were primarily driven by the selective effects of altered diet composition.

Functional prediction based on PICRUSt2 and FAPROTAX consistently indicated that the core functional profile of the rumen microbiota remained relatively stable across different treatment groups. Functions related to carbohydrate metabolism, chemoheterotrophy, and fermentation were dominant, suggesting high metabolic stability and functional redundancy within the rumen microbial ecosystem. On this basis, with increasing substitution levels of *Pennisetum giganteum* silage, fiber-degradation-related functions (e.g., cellulose and xylan degradation) showed an increasing trend. This result was generally consistent with the observed shifts in fiber-degrading microbial communities. Previous studies have demonstrated that high-fiber diets can promote the enrichment of fiber-degrading microorganisms and carbohydrate-active enzymes (CAZymes), thereby improving fiber utilization efficiency [44]. In addition, rumen microbiota can adapt to substrate variation through dynamic adjustments in both community structure and functional expression, thereby enhancing the utilization capacity of structural carbohydrates [45].

## 5. Conclusions

In summary, dietary inclusion of *Pennisetum giganteum* silage at different replacement levels was able to maintain functional homeostasis of the rumen microbiota while exerting moderate modulatory effects on key microbial taxa and their predicted functions. Notably, when 50% of whole-plant corn silage was replaced with *Pennisetum giganteum* silage, the rumen microbial community exhibited favorable adaptability, accompanied by improved rumen fermentation characteristics and enhanced functional potential related to fiber degradation. However, this study did not evaluate animal welfare-related indicators or ruminal mucosal histomorphological parameters. Therefore, future studies should incorporate these measurements to more comprehensively elucidate animal welfare status and the adaptive responses of ruminal structure and function to dietary changes.

## Figures and Tables

**Table 1:** Nutrient composition of *Pennisetum giganteum* silage and whole-crop maize silage (dry matter basis).

Item	*Pennisetum giganteum* Silage	Whole-Crop Maize Silage
Dry matter	77.79	69.10
Crude fat	2.21	3.05
Crude protein	8.97	7.48
Organic matter	87.46	93.22
Neutral detergent fiber	61.73	50.61
Acid detergent fiber	38.78	23.20
Calcium	0.18	0.11
Phosphorus	0.18	0.16

Except for dry matter, the content of other nutrients is based on dry matter.

**Table 2:** Composition and nutrient levels of the experimental diets (dry matter basis).

Item	*Pennisetum giganteum* Silage, % Diet				
	0	25%	50%	75%	100%
Ingredients					
whole-crop maize silage	20.00	15.00	10.00	5.00	0.00
maize stover	38.04	37.09	36.08	35.10	34.16
*Pennisetum giganteum* silage	0.00	5.00	10.00	15.00	20.00
Corn meal	25.13	26.49	27.78	29.12	30.49
Wheat bran	0.50	0.33	0.34	0.24	0.05
Cottonseed meal	8.33	8.09	7.80	7.54	7.30
Soybean meal	3.00	3.00	3.00	3.00	3.00
Premix ^1^	3.00	3.00	3.00	3.00	3.00
Sodium bicarbonate	1.00	1.00	1.00	1.00	1.00
Salt	1.00	1.00	1.00	1.00	1.00
Total	100.00	100.00	100.00	100.00	100.00
Nutrient level, % of DM ^2^					
Gross Energy(MJ/kg)	17.54	17.50	17.49	17.16	17.26
Crude protein	10.03	10.34	9.22	9.63	9.82
Organic matter	91.06	91.14	90.08	89.89	90.00
Neutral detergent fiber	41.81	38.33	40.62	36.32	39.10
Acid detergent fiber	23.51	21.76	24.52	21.24	22.51
Crude fat	2.42	2.41	2.39	2.19	2.26
Calcium	0.50	0.53	0.61	0.70	0.59
Phosphorus	0.17	0.20	0.21	0.22	0.20

^1^ Per kilogram of premix: vitamin A, 66,000–200,000 IU; vitamin D_3_, 25,000–80,000 IU; vitamin E, ≥500 mg; Fe (as ferrous sulfate), 1250–15,000 mg; Cu (as copper sulfate), 280–600 mg; Zn (as zinc sulfate), 1400–2400 mg; Mn (as manganese sulfate), 1100–3000 mg; Se (as sodium selenite), 7–13 mg. ^2^ Nutritional levels were measured values.

**Table 3:** Effects of Different Proportions of *Pennisetum giganteum* Silage on Rumen Fermentation Parameters in Beef Cattle.

Items	*Pennisetum giganteum* Silage, % Diet					SEM	*p*-Value		
	0	25%	50%	75%	100%		Trt	L	Q
pH	6.618	7.040	6.945	6.822	6.892	0.061	0.259	0.443	0.155
NH_3_-N (mg/dL)	4.523 ^b^	4.742 ^b^	8.540 ^a^	8.235 ^a^	8.405 ^a^	0.553	0.014	0.002	0.293
VFAs (mmol/L)	34.890	33.877	42.880	36.800	32.169	1.381	0.121	0.783	0.049
Acetate (mmol/L)	25.206	24.281	30.221	26.427	23.244	0.960	0.179	0.783	0.075
Propionate (mmol/L)	5.137	5.619	6.596	5.923	4.958	0.241	0.178	0.973	0.025
Butyrate (mmol/L)	3.351	3.089	3.881	3.736	2.903	0.128	0.090	0.774	0.053
Acetate/Propionate	4.607	4.332	4.354	4.490	4.661	0.050	0.167	0.447	0.020

^a,b^ Different superscripts indicate significant differences within a row (*p* < 0.05). SEM is the pooled standard error between five groups; the *p*-value indicates significance. Trt, treatment effect; L, linear; Q, quadratic.

**Table 4:** Comparison of Alpha Diversity Indices of the Rumen Microbial Community in Beef Cattle Fed *Pennisetum giganteum* Silage.

Items	*Pennisetum giganteum* Silage, % Diet					SEM	*p*-Value		
	0	25%	50%	75%	100%		Trt	L	Q
Feature	2846.170	2821.000	2901.330	2972.000	2777.330	31.243	0.337	0.952	0.189
ACE	2881.478	2858.912	2939.014	2999.796	2808.279	31.677	0.375	0.980	0.187
Chao1	2855.096	2831.948	2911.757	2978.803	2788.668	31.151	0.358	0.949	0.191
Simpson	0.998	0.998	0.998	0.998	0.998	<0.001	0.937	0.495	0.931
Shannon	10.222	10.238	10.273	10.317	10.203	0.0312	0.825	0.861	0.375
PD_whole_tree	30.788	29.670	29.565	30.066	30.909	0.568	0.928	0.862	0.376
Coverage	0.998	0.998	0.998	0.999	0.999	<0.001	0.535	0.262	0.676

**Table 5:** Effects of *Pennisetum giganteum* silage on the relative abundance of rumen microbiota at the phylum level in beef cattle.

Items	*Pennisetum giganteum* Silage, % Diet					SEM	*p*-Value		
	0	25%	50%	75%	100%		Trt	L	Q
Actinobacteriota	0.010	0.017	0.005	0.010	0.009	0.002	0.557	0.571	0.979
Bacteroidota	0.457	0.436	0.494	0.432	0.464	0.009	0.229	0.861	0.850
Cyanobacteria	0.007	0.005	0.010	0.010	0.007	0.001	0.156	0.501	0.175
Desulfobacterota	0.004	0.004	0.004	0.006	0.004	<0.001	0.284	0.298	0.531
Fibrobacterota	0.007	0.010	0.010	0.009	0.004	0.001	0.245	0.350	0.037
Firmicutes	0.406	0.418	0.357	0.425	0.405	0.012	0.435	0.951	0.512
Patescibacteria	0.020	0.026	0.025	0.021	0.024	0.001	0.680	0.842	0.571
Proteobacteria	0.021	0.022	0.026	0.028	0.036	0.002	0.256	0.033	0.520
Spirochaetota	0.028	0.028	0.028	0.025	0.017	0.002	0.172	0.039	0.161
Verrucomicrobiota	0.033 ^ab^	0.021 ^c^	0.035 ^a^	0.028 ^abc^	0.022 ^bc^	0.002	0.034	0.211	0.563

^a,b,c^ Different superscripts indicate significant differences within a row (*p* < 0.05). SEM is the pooled standard error between five groups; the *p*-value indicates significance. Trt, treatment effect; L, linear; Q, quadratic.

**Table 6:** Effects of *Pennisetum giganteum* silage on the relative abundance of rumen microbiota at the genus level in beef cattle.

Items	*Pennisetum giganteum* Silage, % Diet					SEM	*p*-Value		
	0	25%	50%	75%	100%		Trt	L	Q
*Prevotella*	0.181	0.179	0.193	0.175	0.198	0.006	0.692	0.483	0.740
*uncultured_rumen_bacterium*	0.107	0.096	0.117	0.106	0.099	0.003	0.100	0.742	0.251
*Rikenellaceae_RC9_gut_group*	0.082	0.074	0.080	0.072	0.069	0.003	0.459	0.129	0.854
*Christensenellaceae_R_7_group*	0.053	0.053	0.036	0.068	0.053	0.004	0.264	0.643	0.564
*NK4A214_group*	0.043	0.040	0.030	0.044	0.041	0.003	0.559	0.990	0.305
*unclassified_F082*	0.041	0.043	0.041	0.035	0.038	0.001	0.408	0.159	0.770
*unclassified_Bacteroidales_RF16_group*	0.033	0.021	0.045	0.036	0.033	0.003	0.306	0.518	0.631
*Ruminococcus*	0.029	0.027	0.023	0.026	0.024	0.002	0.828	0.382	0.665
*Treponema*	0.025	0.026	0.024	0.022	0.014	0.002	0.164	0.034	0.166
*Prevotellaceae_UCG_003*	0.021 ^b^	0.024 ^ab^	0.027 ^a^	0.019 ^b^	0.021 ^b^	0.001	0.019	0.316	0.031
Others	0.385	0.411	0.384	0.399	0.410	0.005	0.350	0.340	0.770
Unknown	0.001	0.006	0.000	0.000	0.001	0.001	0.459	0.448	0.651

^a,b^ Different superscripts indicate significant differences within a row (*p* < 0.05). SEM is the pooled standard error between five groups; the *p*-value indicates significance. Trt, treatment effect; L, linear; Q, quadratic.

## Data Availability

The raw sequencing reads were deposited into the NCBI Sequence Read Archive (SRA) data: PRJNA1450945.
